# Lower body energy generation, absorption, and transfer in youth baseball pitchers

**DOI:** 10.3389/fspor.2022.975107

**Published:** 2022-09-21

**Authors:** Moira K. Pryhoda, Michelle B. Sabick

**Affiliations:** Human Dynamics Laboratory, Department of Mechanical and Materials Engineering, University of Denver, Denver, CO, United States

**Keywords:** biomechanics, baseball pitching, power, energy, sport performance

## Abstract

An efficient baseball pitch will produce a high-velocity ball while minimizing the risk of injury to the pitcher. This study quantified ground reaction forces and lower body power during the entire pitching motion of youth baseball pitchers to investigate how developing athletes generate and transfer energy from lower limbs to the throwing arm. These data provide a foundation for comparing youth pitching strategy and mechanics to optimal throwing mechanics and may aid in developing appropriate training suggestions for this age group. Full-body three-dimensional (3D) motion capture and force platform data were collected on 23 youth pitchers performing fastballs thrown for strikes. Youth pitchers within this study used a “controlled drop” strategy in which the COM was lowered during the stride phase followed by a weak forward drive motion. Ground reaction forces (GRFs) indicate that the drive leg propels the center of mass (COM) toward the home plate while the stride leg braking force contributes to power generation up the kinetic chain. The stride hip generates energy assisting in energy flow up the kinetic chain as well as the creation of a stable base to rotate the trunk about. The lumbosacral joint generates the most energy of any joint studied, facilitating energy flow up the kinetic chain and underscoring the importance of core strength and coordination in proper pitching mechanics.

## Introduction

The goal of an effective baseball pitch is to generate and transfer energy through the body to deliver an accurate, high-velocity ball over the home plate. Each linked segment in the kinetic chain has unique kinematic patterns and holds the capacity to passively and actively generate, absorb, and/or transfer energy. Proper pitching technique is dependent on the timing of key events and activation of the musculature crossing each joint during the pitch cycle [1–3]. An optimal pitching technique will not only maximize pitch velocity [4] but will also minimize injury risk to the pitcher [5] for a given level of effort. Currently, up to 50% of youth baseball players report upper extremity pain during the season [6, 7], and the rate of an elbow injury and surgery has risen in adolescent baseball pitchers so much that it is considered an epidemic [8–10]. The most common age for ulnar collateral ligament (UCL) reconstruction is now only 15–19 years old [11]. Strategies to limit pitches per game and eliminate pitch types associated with increased incidence of shoulder and elbow pain have been suggested to decrease injury risk [6], but effective mechanics-based strategies using biomechanical assessments for injury rate reduction are also needed.

**Figure 1:**
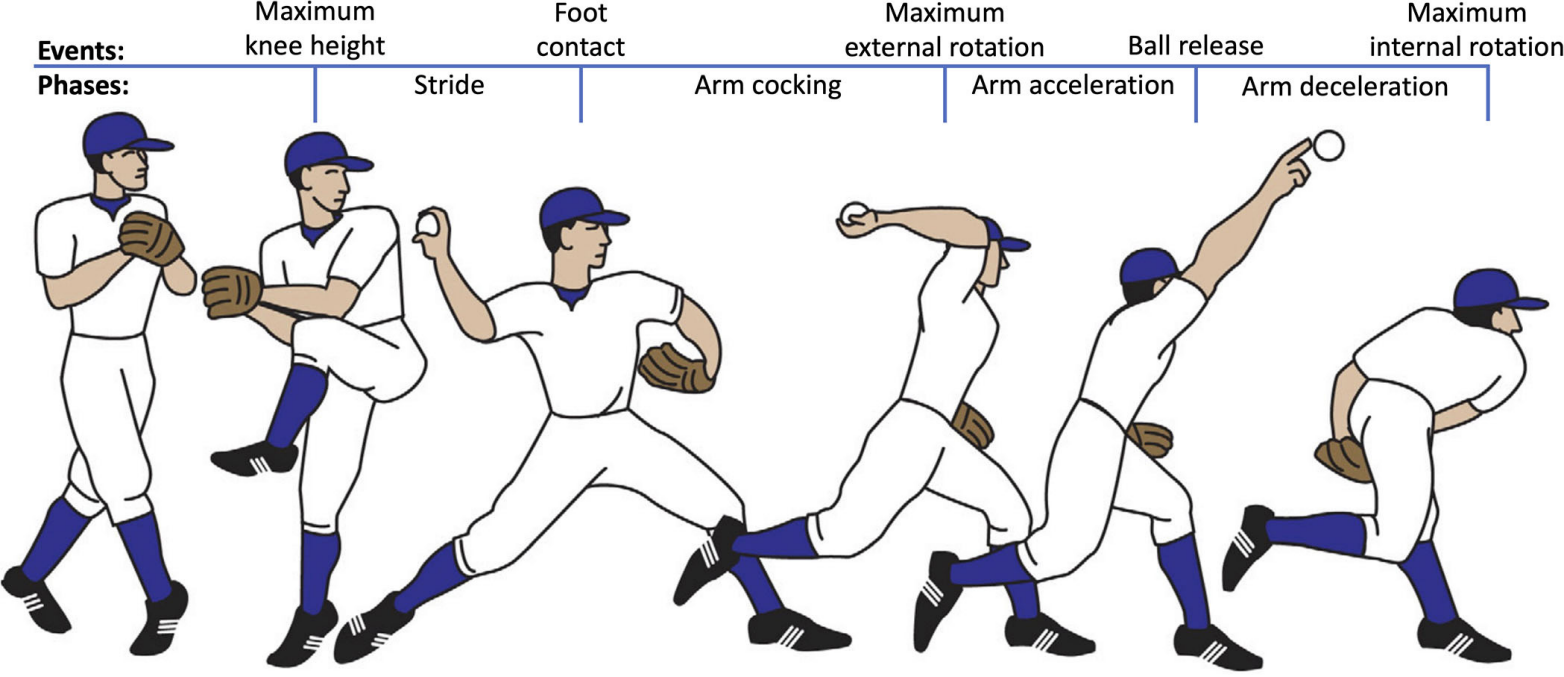
Events and phases of the baseball pitch used in biomechanical analysis. Modified from Braun et al. [16].

Biomechanical assessments of the baseball pitch have historically used timing and magnitude of kinematic events as the primary means to assess performance [12–15]. During the wind-up, weight is transferred to the drive (back) leg and the stride (front) leg lifts forcing the center of mass over the drive leg before maximum knee height (MKH, see Figure 1). During the stride phase (MKH to stride foot contact; SC), the pitcher pushes the drive leg against the ground to generate a forward-directed ground reaction force (GRF) [17, 18]. In the arm cocking phase (SC to maximum external rotation; MER), the stride leg serves as a stable base about which to rotate. Stride leg knee extension and pelvis rotation end shortly after foot strike allowing energy to be transferred up the kinetic chain from the lower extremities into the trunk [17, 18]. The upper body rotates allowing the throwing arm to achieve maximum external rotation. In the arm acceleration phase (MER to ball release; BR), the torso rotates and flexes both forward and laterally to assist with kinetic chain energy transfer to the throwing arm [17, 18]. The stride knee extends to stabilize the pelvis and transfer energy up from the lower limbs [17]. The shoulder internally rotates, and the elbow extends to transfer energy to the hand and ultimately the ball at release. Finally, during the arm deceleration phase (BR to maximum internal rotation; MIR), the shoulder reaches maximum internal rotation and the torso tilts forward as the muscles of the trunk and arm work to slow down the arm and reduce joint loading [17, 18].

Biomechanics studies identifying the timing and magnitude of specific kinematic features of the pitch often conclude that the solution to improper kinematics is increased lower extremity and core strength and that increased energy generation of the lower limbs will aid in both increased throwing velocity and decreased upper extremity injury rates [1, 4, 19, 20]. Studies investigating pitching kinetics indirectly support this assertion [19, 21–24]. For example, higher velocity pitchers have been found to have greater propulsive and braking forces, pelvis and trunk angular velocity, and drive hip abduction [19, 22]. Aguinaldo et al. [21] found that improper trunk timing causes the throwing arm to contribute more to the throw, increasing the risk for overuse injury. Power and energy flow analyses may provide more direct measures of how pitching mechanics relate to performance and injury risk; however, work has largely focused on the upper extremities [23, 24] rather than on the lower extremities and trunk.

Theoretical models of the roles of the lower limbs in pitching provide a starting point for how we may expect energy to flow during the pitch [25, 26]. There are two leading theories among coaches describing the appropriate use of the drive leg in pitching: [1] drop and drive, and [2] controlled fall. The drop and drive theory suggests that pitchers lower their center of mass in the stride phase followed by propelling their body forward using the drive leg musculature before stride foot contact. The controlled fall theory suggests that the drive leg joints do not directly contribute power, and instead focuses is placed on using the lower extremities to produce a strong and sufficient base of support. Elliot et al. [1] used ground reaction forces to determine which theory is prevalent in professional baseball pitchers and found that the drive leg in pitching was characterized as a combination of controlled fall and max effort drive based on peak braking and propulsive GRFs. In terms of the stride leg, it is widely accepted that pitchers land on the stride leg with full body weight to redirect energy up the kinetic chain and to provide a stable base upon which to rotate [27]. The role of the trunk is then to transfer the power generated in the drive and stride legs while generating additional power to be transferred into the throwing shoulder and arm [2, 28]. Further biomechanical analysis of the lower body will increase understanding of the roles of each joint during the pitch motion.

The purpose of this study was to analyze ground reaction forces and lower body power continuously during the entire pitching motion of youth baseball pitchers to determine what lower limb energy flow strategy developing athletes use. We hypothesized that there would be significant increases in peak joint torque power and significant differences in the timing of peak powers in each subsequent joint up the kinetic chain. These data will provide a foundation for future comparisons to optimal throwing mechanics and will aid in establishing developmentally appropriate suggestions for improving pitch mechanics to reduce throwing arm injury while maintaining or increasing pitch velocity.

## Methods and materials

### Participants

Male baseball pitchers (*n* = 23, mass = 46.19 ± 11.71 kg, height = 1.56 ± 0.12 m, BMI = 18.79 ± 2.55) between the ages of 9–13 from youth baseball teams in the greater Milwaukee area participated in this study. Pitchers were eligible for participation if they had at least 1 year of pitching experience, utilized an “overhead” pitching motion (see Figure 1), had no previous injury to the throwing arm requiring surgery or removal from play for at least 3 months, had no current injury affecting the ability to pitch, and presented with no chronic arm pain or soreness.

### Apparatus

**Figure 2:**
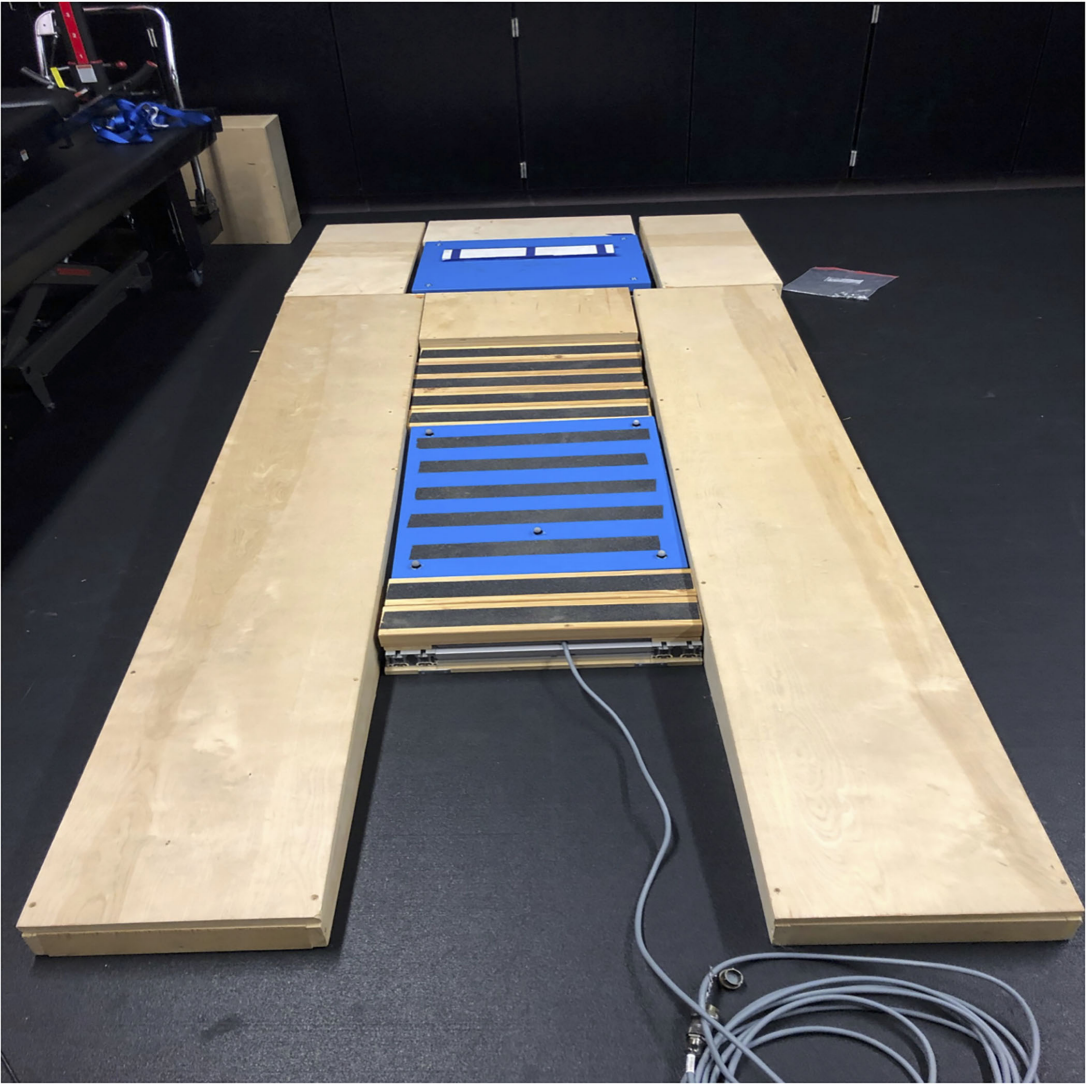
Custom major league baseball regulation size pitching mound with two embedded force platforms.

Data were collected using two force platforms (50 cm × 60 cm, Kistler) embedded in a custom-built, Major League Baseball regulation size pitching mound (Figure 2). Ground reaction forces were recorded at 1,000 Hz and a 14-camera motion capture system (Vicon) sampled motion data at 250 Hz. One force plate was placed under the pitching rubber and mounted onto a rigid steel frame to ensure no signal loss due to frame deformation. The second force plate was mounted in the landing area of the mound on a moveable track to enable position adjustment based on the stride length of the pitcher. A hanging target surrounded by a net was placed at the regulation distance for Little League Baseball (14 m) from the pitching rubber.

### Procedure

Pitchers were outfitted with a full body marker set including an upper extremity marker set consistent with International Society of Biomechanics (ISB) recommendations [29], and a full lower extremity marker set for a combined total of 32 individual markers and seven marker clusters [30]. Individual markers were secured to anatomical landmarks with double-sided tape, and marker clusters were secured with flexible adhesive bandages. Marker clusters were placed on both thighs, both shanks, and the upper arm, forearm, and hand of the throwing arm. These were included for joint reconstruction purposes in addition to markers placed on anatomical landmarks. Subjects were instructed to warm up by stretching and throwing as they would for a typical game. Following the warm-up, the subject was instructed to throw a total of 15 maximum effort fastballs toward the target. The pitcher was given feedback following each pitch regarding the recorded velocity, accuracy, and whether it was considered a strike or ball. Pitches were considered a strike if the ball hit the hanging target and were considered a ball if the ball hit the net surrounding the hanging target.

### Data processing

The three fastest pitches thrown for strikes by each pitcher were chosen for further analysis. The full dataset of three pitches from all 23 pitchers had an average ball speed of 27.07 ± 3.91 m/s. All kinematic and kinetic analyses were carried out in Visual 3D (Version v6, C-Motion, Inc, Germantown, MD, USA). Data were filtered with a 4th order zero phase lag Butterworth filter with cutoff frequencies of 18 Hz and 300 Hz for marker trajectory and force plate data, respectively. Joint angles, angular velocities, and joint moments of the lower extremities (ankles, knees, and hips) were calculated by transforming the distal segment coordinate system to that of the proximal segment using a Cardan sequence of rotations. For the purposes of power and energy calculations an artificial joint construct, the lumbosacral joint, was defined as a simplification of the connection between the pelvis and the trunk. Joint angles, angular velocities, and joint moments of the lumbosacral joint were defined by expressing the pelvis coordinate system with respect to the trunk coordinate system. Joint torque power [JTP; Equation [1]] was calculated at all joints by summing the dot product of the proximal segment external joint moment and its angular velocity (segment torque power of the proximal segment; STP_p_) with the dot product of the distal segment external joint moment and its angular velocity (segment torque power of the distal segment; STP_d_) [12, 31]. All joint moments and angular velocities were calculated as three-dimensional vectors in all three planes of motion, the dot products of which produce a scalar JTP. After calculating power in Watts, data were normalized to body weight times height prior to analysis.


(1) $$\begin{array}{l} JTP\;=\;{STP_{p}\;+\;STP_{d}\;=\;M}_{p}\;•\;w_{p}\;+\;M_{d}\;•\;w_{d} \end{array}$$

All data were time-normalized from MKH (0%) to MIR (100%) events detected during the pitch. The three pitches analyzed from each of the 23 pitchers were used to create group means and standard deviations as a function of time for each variable of interest. Peak values from time series data are presented as group mean ± standard deviation (SD). Peak JTP magnitudes and timing are considered significantly different with *p* < 0.05.

## Results

### Ground reaction force

**Figure 3:**
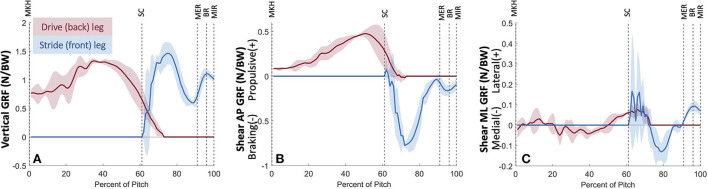
Mean ± SD ground reaction forces along the vertical **(A)**, anterior-posterior **(B)**, and mediolateral **(C)** axes for the three fastest pitches thrown for strikes from 23 developmental-aged pitchers for the duration of the pitch from maximum knee height (MKH) to maximum internal rotation (MIR).

Vertical GRF was the largest force component, with the drive leg producing peak vertical GRF of 1.33 ± 0.04 N/BW at 35% of the pitch and the stride leg producing peak vertical GRF of 1.47 ± 0.18 N/BW at 75% of the pitch (Figure 3). Anterior–posterior (AP) shear force in the direction of pitch (Figure 3) indicates that the drive leg creates a 0.48 ± 0.03 N/BW propulsive force peaking before SC at 50% of the pitch. Following SC, the stride leg creates a braking force with a peak magnitude of 0.77 ± 0.02 N/BW at 72% of the pitch. There is little mediolateral (ML) shear force production for the duration of the pitch (Figure 3). The drive leg creates small lateral and medial shear forces during the stride phase. The stride leg has two low magnitude lateral shear peaks following SC, a low magnitude medial shear force peaking at 0.13 ± 0.04 N/BW at 79% of the pitch, and a lateral shear force peaking at 0.09 ± 0.03 N/BW at MER (97% of the pitch).

### Drive leg power

**Figure 4:**
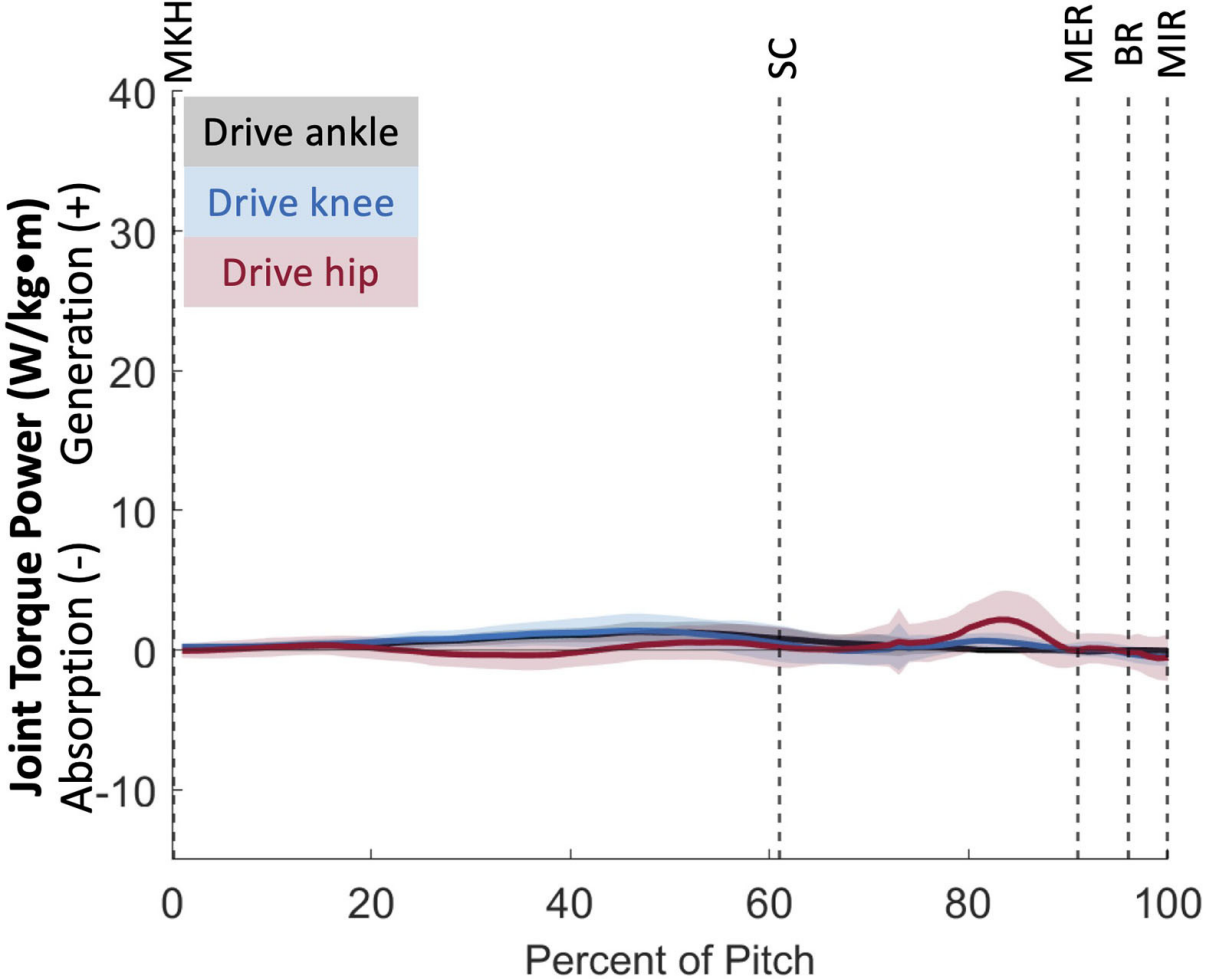
Mean ± SD joint torque power for the joints of the drive leg (black = ankle, blue = knee, red = hip) for the duration of the pitch from maximum knee height (MKH) to maximum internal rotation (MIR).

**Figure 5:**
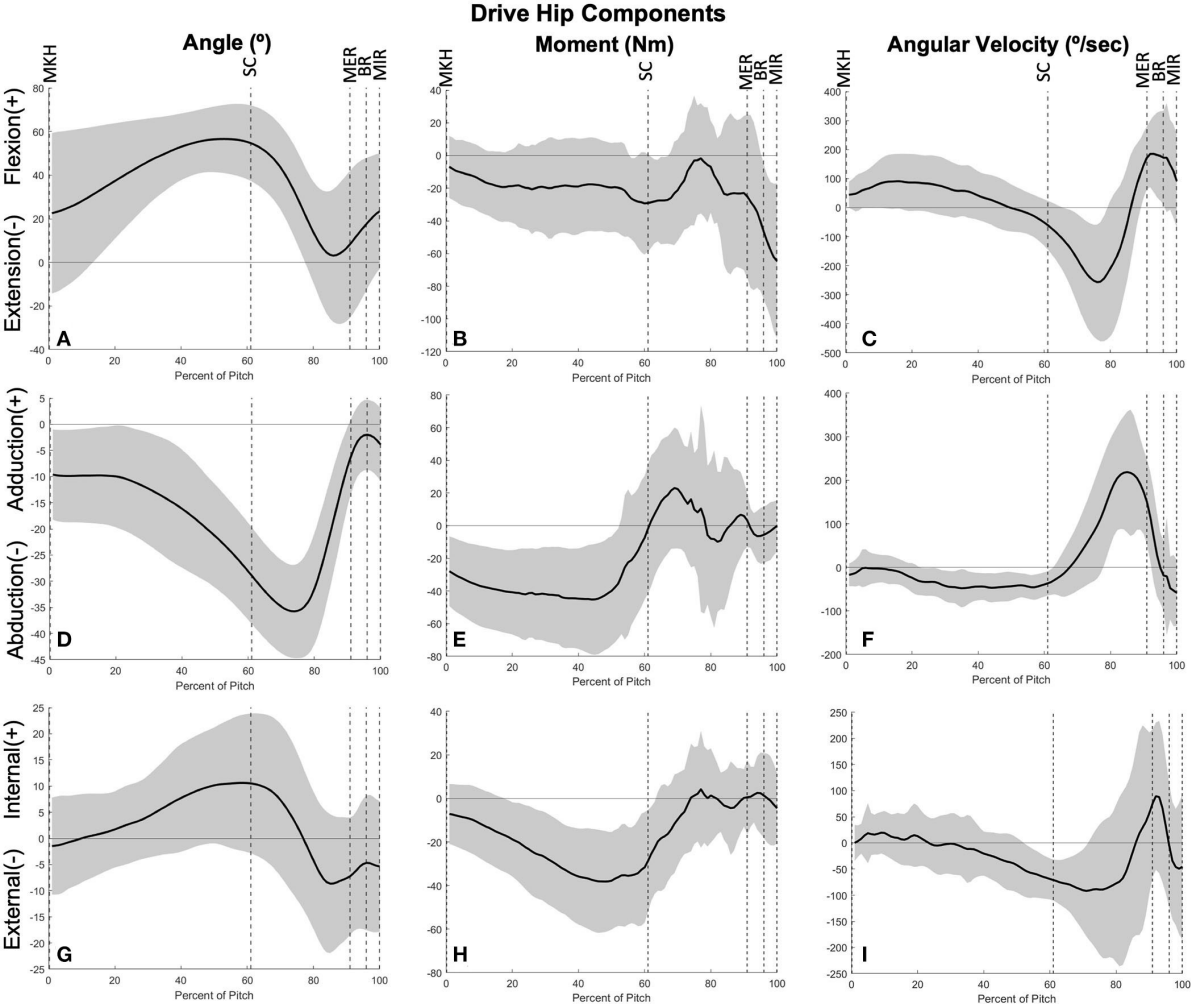
Mean ± SD joint angle, joint moment, and joint angular velocity for the drive hip in extension/flexion **(A–C)**, ab/adduction **(D–F)**, and internal/external rotation **(G–I)** for the duration of the pitch from maximum knee height (MKH) to maximum internal rotation (MIR).

**Figure 6:**
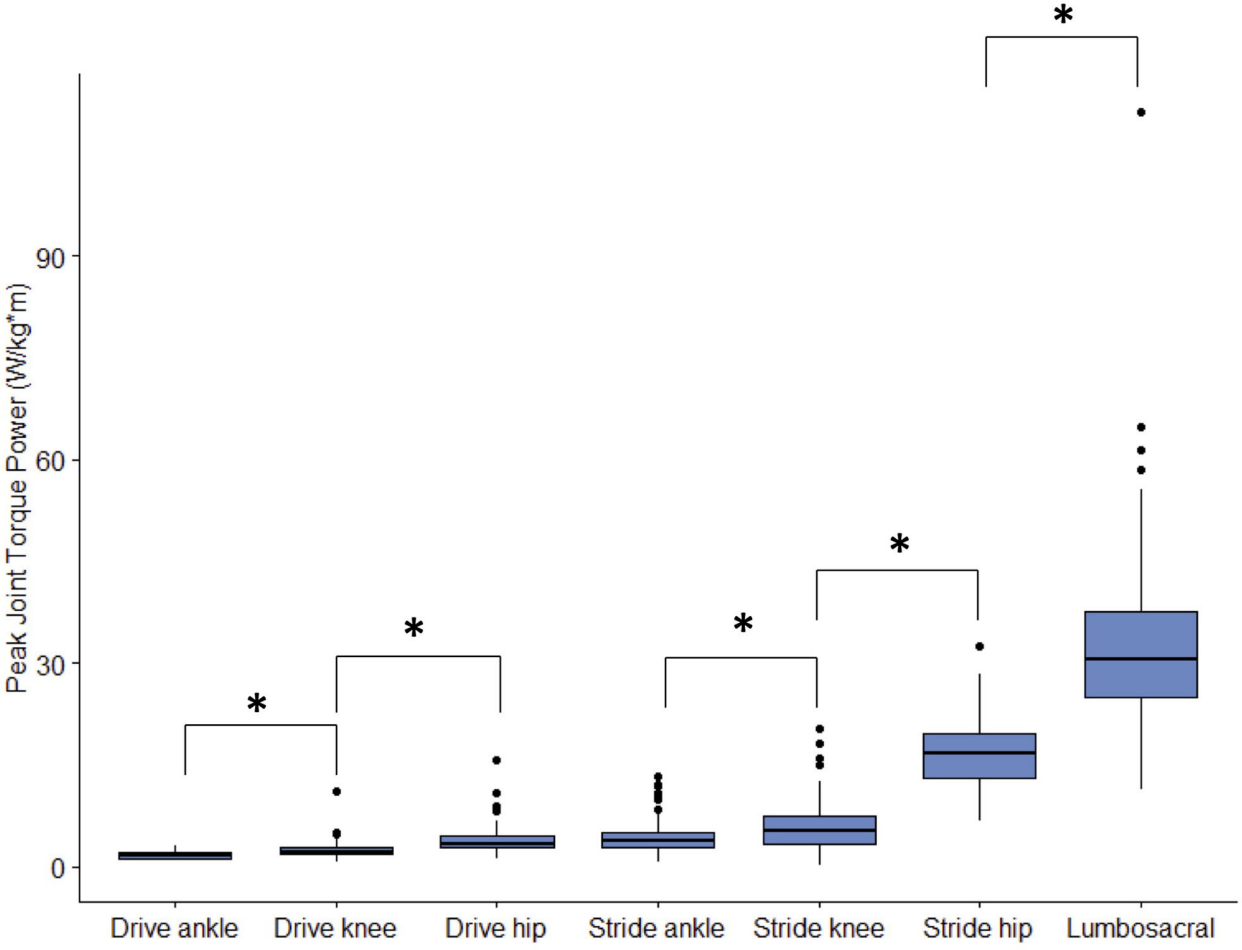
Peak joint torque power magnitude for each lower body joint from the three fastest pitches thrown for strikes from 23 developmental-aged pitchers. “*” indicates statistically significant differences with *p* < 0.05.

**Figure 7:**
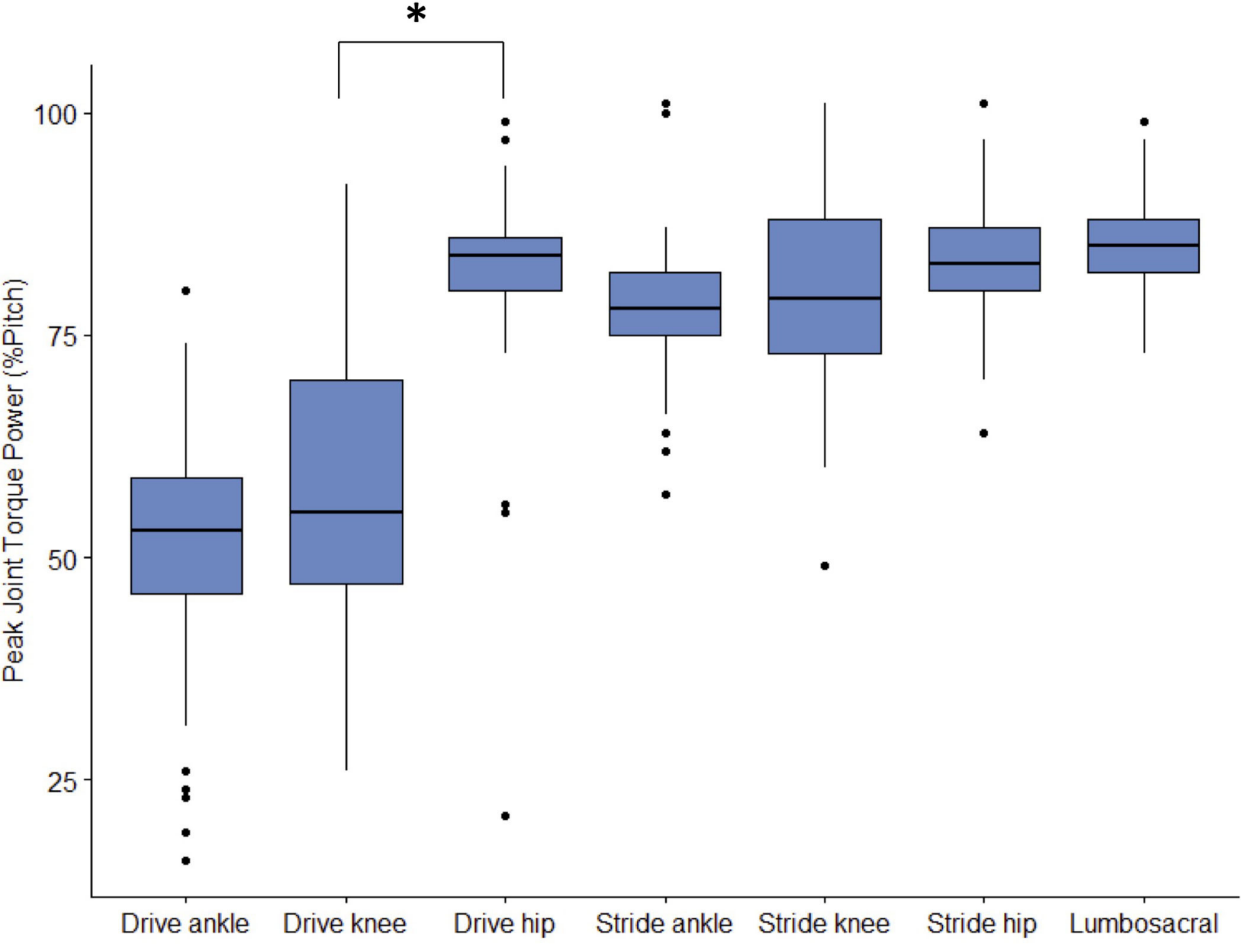
Peak joint torque power percent of pitch cycle for each lower body joint from the 3 fastest pitches thrown for strikes from 23 developmental-aged pitchers. “*” indicates statistically significant differences with *p* < 0.05.

The drive leg joints generated minimal power (Figure 4) in comparison to stride leg and lumbosacral joints. The drive ankle and knee have slight power generation during the stride phase (1.80 ± 0.60 W/kg^*^m at 52 ± 14% and 2.47 ± 1.36 W/kg^*^m at 57 ± 16%, respectively). The power generation of the drive ankle is created by extension, eversion, and abduction moments, while the power generation of the drive knee has contributions from extension, abduction, and internal rotation moments. The drive hip generates a small amount of power shortly before MER in the arm cocking phase (4.10 ± 2.24 W/kg^*^m; 82 ± 10% of the pitch), which appears to be generated using the extensors (Figure 5). Peak JTP of the drive knee is significantly greater (*p* = 0.001) than that of the drive ankle and the peak JTP of the drive hip is significantly greater (*p* < 0.001) than that of the drive knee (Figure 6), indicating that the joints of the drive leg sequentially generate more power up the kinetic chain during the stride and arm cocking phases. The timing of peak JTP was not significantly different between the drive ankle and drive knee, but peak JTP of the stride hip was significantly later during the pitch cycle compared to peak JTP of the stride knee (*p* < 0.001; Figure 7).

### Stride leg power

**Figure 8:**
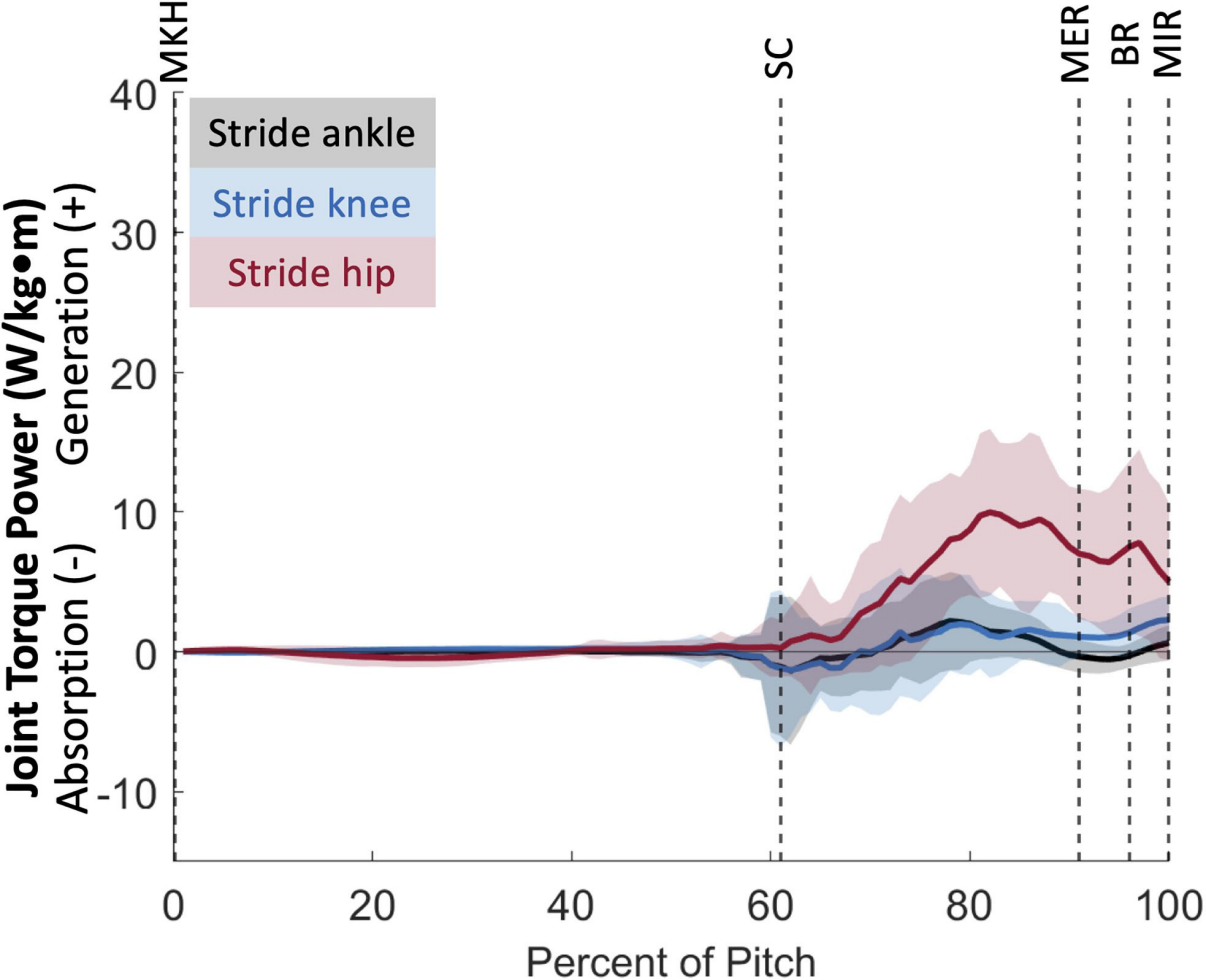
Mean ± SD joint torque power for the joints of the stride leg (black = ankle, blue = knee, red = hip) for the duration of the pitch from maximum knee height (MKH) to maximum internal rotation (MIR).

**Figure 9:**
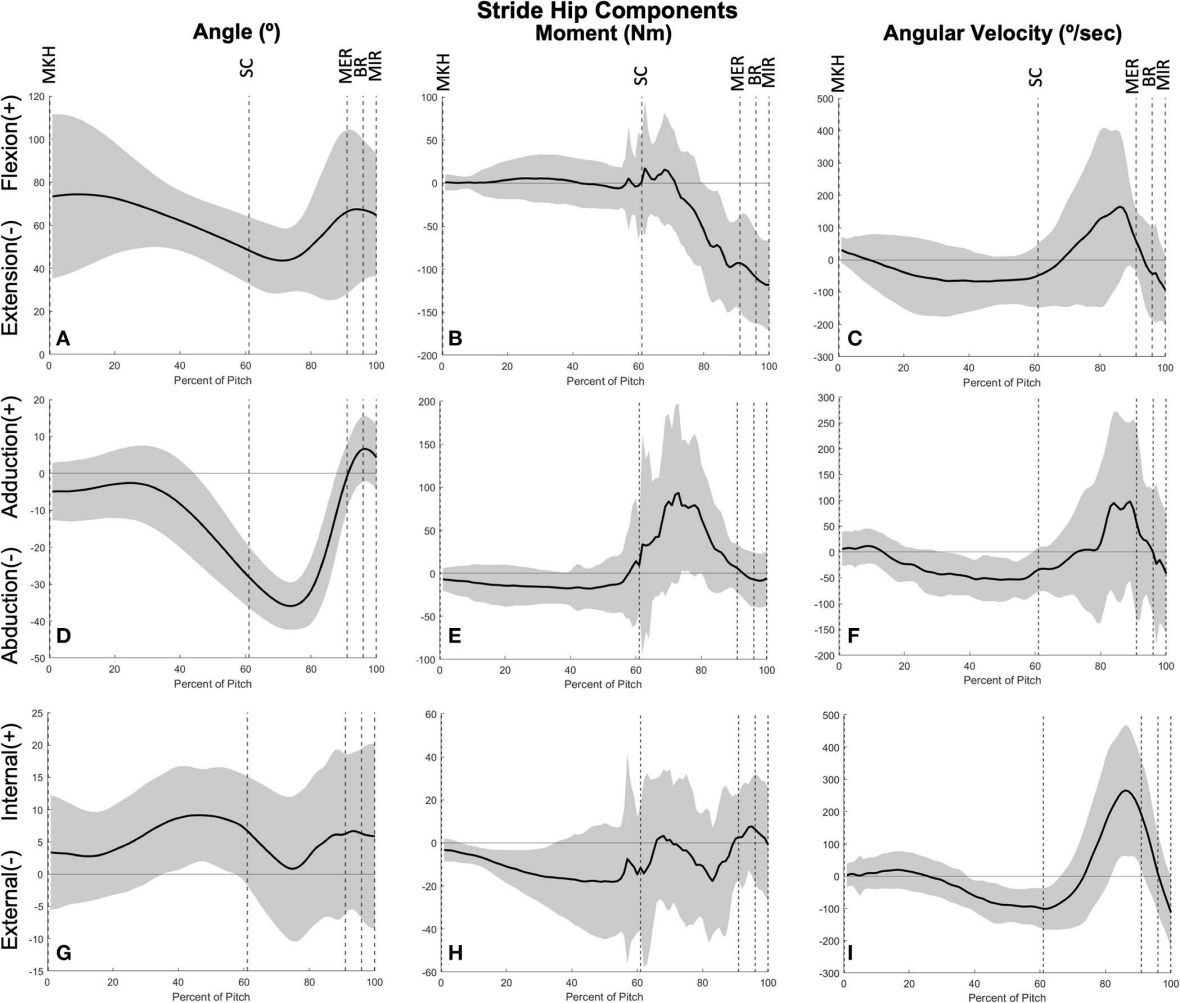
Mean ± SD joint angle, joint moment, and joint angular velocity for the stride hip in extension/flexion **(A–C)**, ab/adduction **(D–F)**, and internal/external rotation **(G–I)** for the duration of the pitch from maximum knee height (MKH) to maximum internal rotation (MIR).

The joints of the stride leg contribute to power generation much more than those of the drive leg. The stride ankle and knee have slight power generation (4.57 ± 2.75 W/kg^*^m at 78 ± 8% and 6.05 ± 3.95 W/kg^*^m at 81 ± 12%, respectively) in the arm cocking phase (Figure 8). This power is generated by ankle plantar flexors and knee flexors as the ankle and knee are extended during this phase, with the knee reaching peak extension angular velocity at BR. The ankle continues to generate power for the duration of the pitch. The stride hip generates power peaks during the arm cocking phase shortly before MER at 84 ± 7% of the pitch (16.94 ± 5.48 W/kg^*^m). This power generation appears to have contributions from musculature surrounding the hip in all three planes of motion. The largest contribution is from the hip extensors, with smaller hip adductor and hip external rotator contributions (Figure 9). The peak hip flexion, adduction, and internal rotation velocities all occur during the arm cocking phase just before MER (Figure 9). Peak JTP of the stride knee was significantly greater than that of the stride ankle (*p* = 0.002) and the peak JTP of the stride hip was significantly greater than that of the stride knee (*p* < 0.001; Figure 6), indicating that like the drive leg, the magnitude of power generation increases up the kinetic chain. Pitch cycle timing of peak JTPs of the stride leg was not significantly different (Figure 7), indicating that JTPs are generated simultaneously.

### Lumbosacral joint power

**Figure 10:**
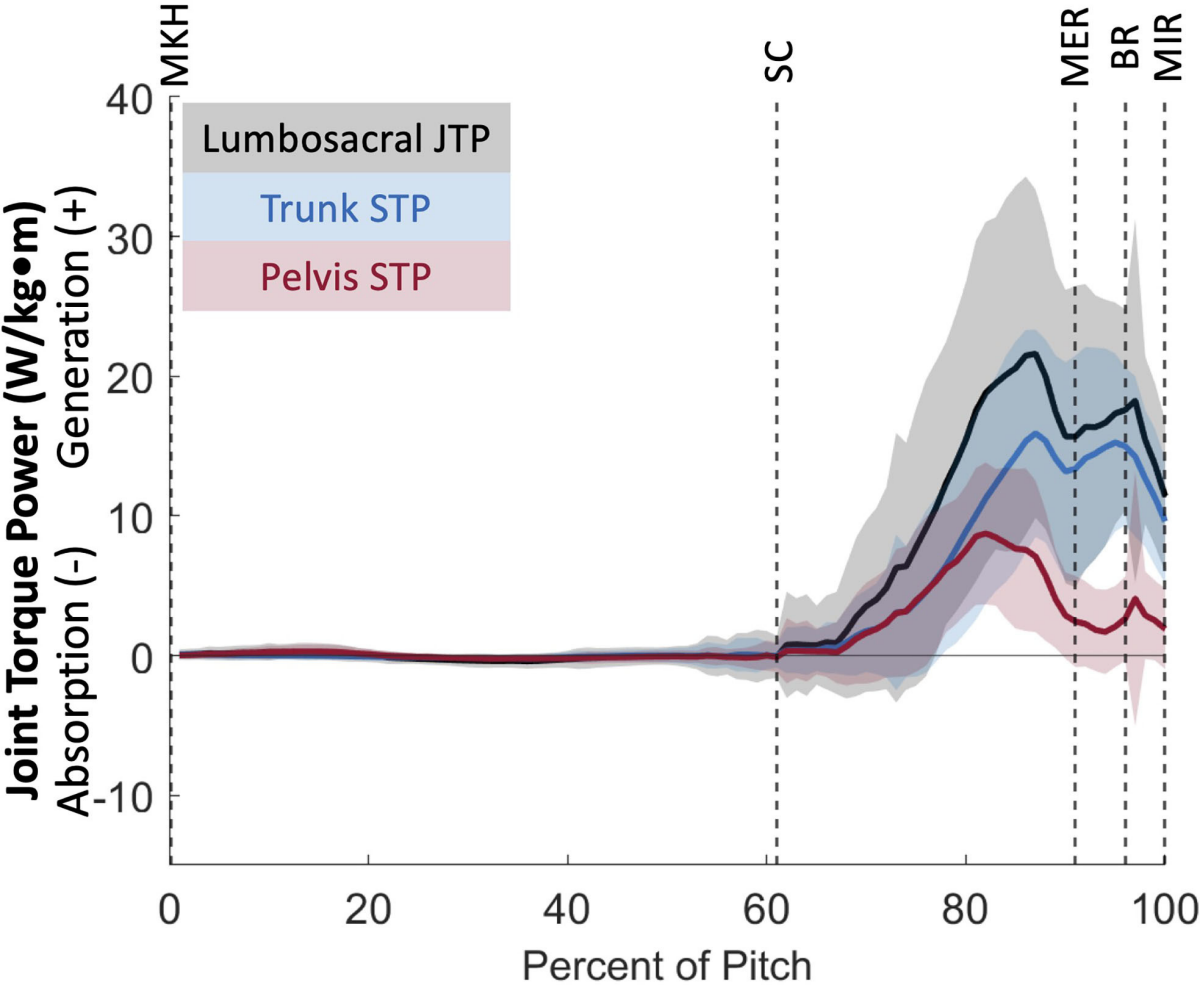
Mean ± SD joint torque power for the lumbosacral joint (black) and its components of trunk segment torque power (blue) and pelvis segment torque power (red) for the duration of the pitch from maximum knee height (MKH) to maximum internal rotation (MIR).

**Figure 11:**
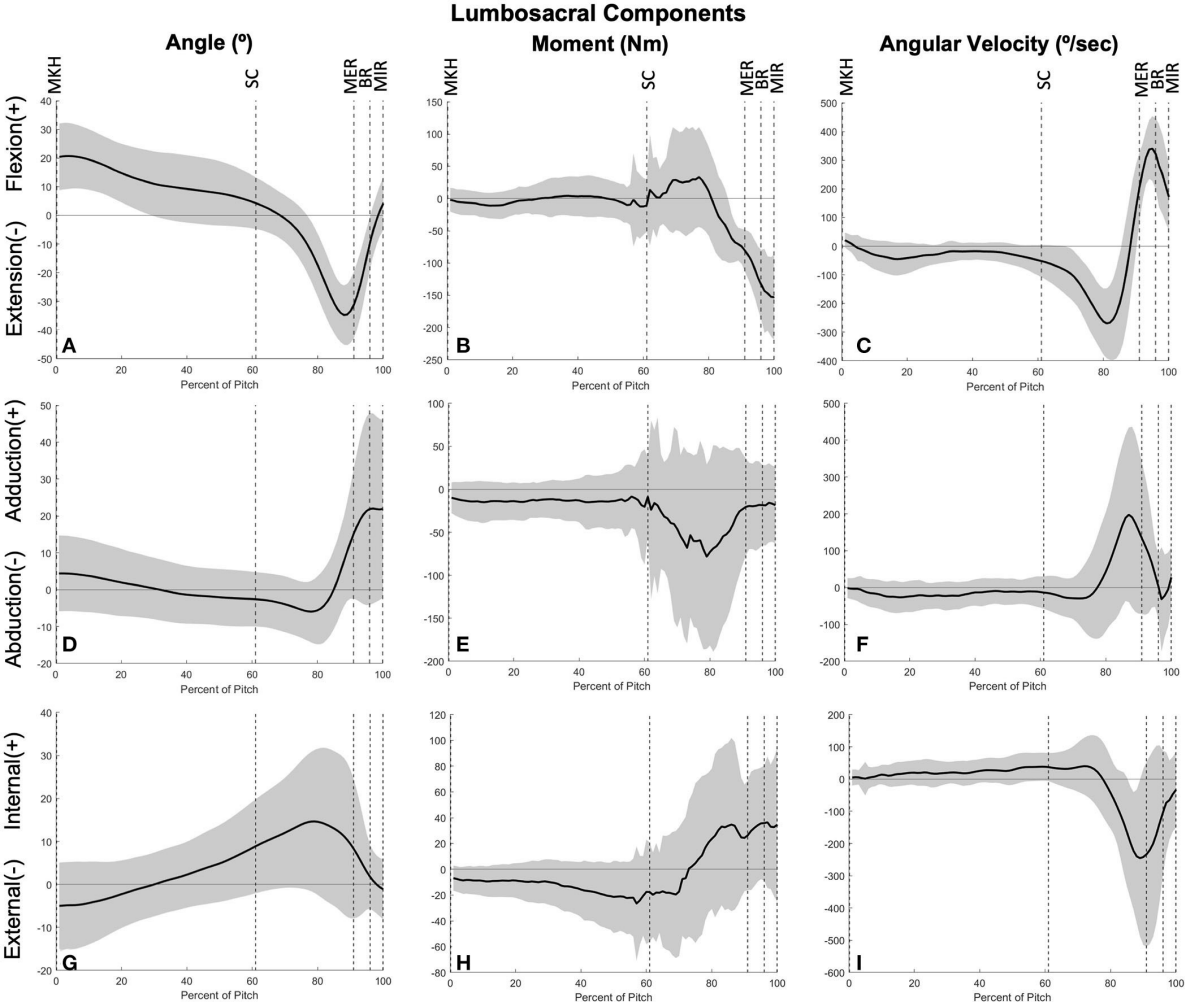
Mean ± SD joint angle, joint moment, and joint angular velocity for the lumbosacral joint in extension/flexion **(A–C)**, ab/adduction **(D–F)**, and internal/external rotation **(G–I)** for the duration of the pitch from maximum knee height (MKH) to maximum internal rotation (MIR).

The lumbosacral (LS) joint generates the most power of any joint studied during the pitch, with a peak power of 32.87 ± 14.62 W/kg^*^m just prior to MER at 85 ± 6% of the pitch and a secondary peak of 18.24 ± 13.04 W/kg^*^m at BR (97% of the pitch; Figure 10). There are no instances of power absorption at this joint. The peak power generation before MER appears to have the largest contribution from LS extensors as the lumbosacral joint reaches peak lateral flexion angular velocity (Figure 11). The power generation at BR is induced by LS extensors and LS internal rotators as well as flexion angular velocity that peaks just before BR (Figure 11). LS JTP magnitude is significantly greater than all lower extremity joints including the stride hip (*p* < 0.001; Figure 6). The timing of LS JTP is not significantly different from the joints of the stride leg and the drive hip (Figure 7).

## Discussion

This study analyzed ground reaction forces and lower body power continuously during the entire pitching motion to understand the lower limb energy flow strategy of youth baseball pitchers. There were significant differences in peak JTP of each subsequent joint in the kinetic chain for both the drive and stride leg. LS JTP was significantly greater than all lower extremity joints. There were no significant differences in the timing of peak JTP in each subsequent joint up the kinetic chain, with the exception of drive hip JTP occurring significantly later than drive knee peak JTP.

### A full pitch strategy

**Figure 12:**
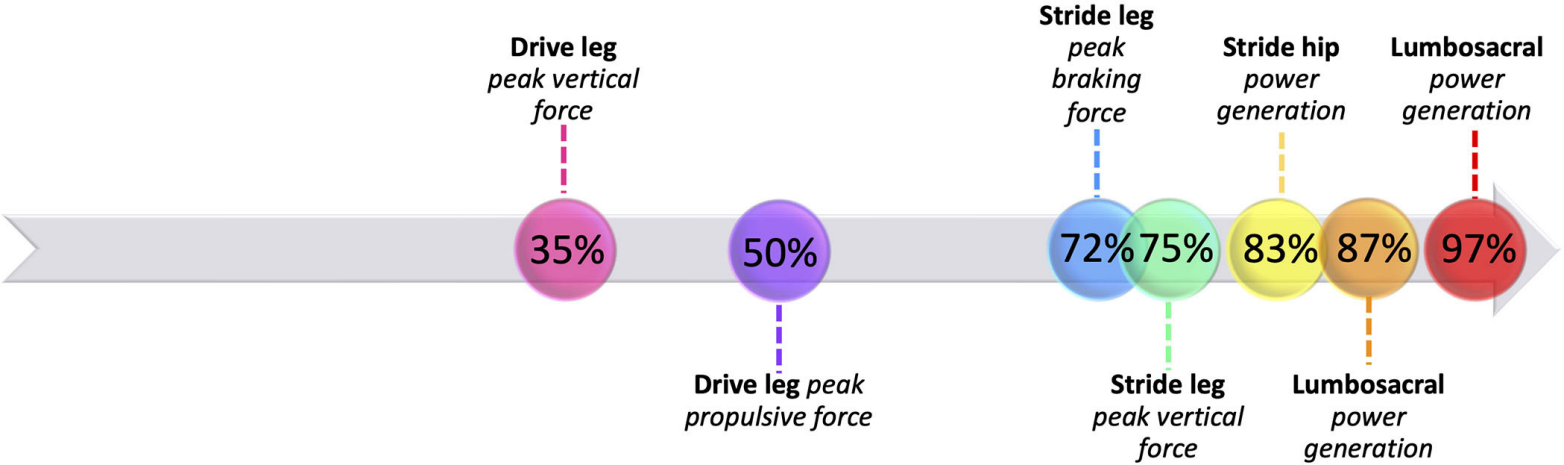
A timeline of the most prominent biomechanical events during the pitch for developmental-aged pitchers.

**Figure 13:**
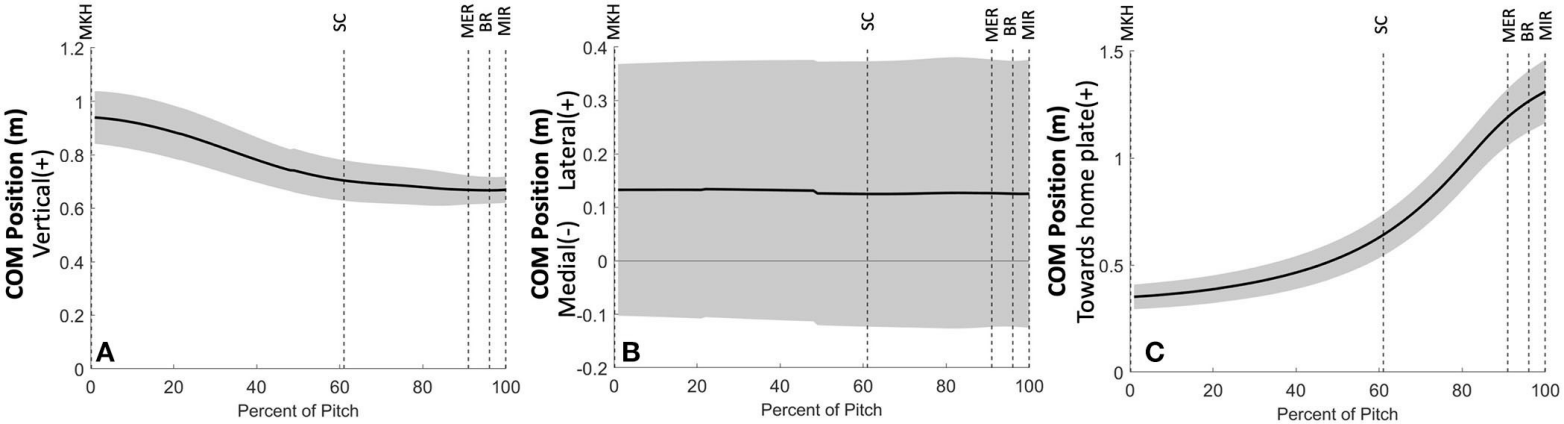
Mean ± SD center of mass position along the vertical **(A)**, mediolateral **(B)**, and anterior-posterior (towards home plate; **C**) global coordinate axes for the 3 fastest pitches thrown for strikes from 23 developmental-aged pitchers for the duration of the pitch from maximum knee height (MKH) to maximum internal rotation (MIR).

Ground reaction forces and joint powers generated and absorbed at the lower body joints define a strategy in which youth pitchers aim to produce a high-velocity pitch (Figure 12). From MKH to approximately 35% of the pitch motion, there are no substantial peak GRF, power generation, or power absorption events. At approximately 35% of the pitch, the drive leg produces peak vertical GRF. Shortly thereafter, drive leg propulsive force peaks at approximately 50% of the pitch, after the center of mass (COM) has substantially lowered through drive leg joint flexion (Figure 13). The peak braking force and peak vertical GRF of the stride leg occur almost simultaneously, and their timing and magnitudes indicate that both components may aid in pelvis deceleration to create a stable base as well as force production through the stride leg as weight transfers largely to the stride leg. For both legs, the vertical and AP shear GRF components appear to be the largest contributors to the total force, which combined with moment arms and segmental angular velocities aid in the creation of joint power. The main contributions of ML shear forces are likely in stabilizing the position of the COM to maintain a forward trajectory (see Figure 13). The small lateral and medial shear forces during the stride phase of the drive leg may be due to foot pronation to shift weight into the ball of the foot aiding in motion toward home plate and/or contributions to balance. Following SC at ~70% of the pitch, the stride leg produces peak braking force to assist in power production at the stride leg joints. This is paired with low magnitude lateral shear peaks which may contribute to stabilizing the base of support (BOS) upon which the body can rotate about as the shoulders square to the home plate. These lateral shear peaks have high between-subjects variability which could be due to differences in technical skill level or a featureless balance strategy. These events are followed by small medial shear peaks produced by the stride leg for BOS stabilization, peak vertical GRF at the stride leg, and power generation at the drive ankle and knee. Soon afterward, the stride hip generates substantial power during the arm cocking phase. Following hip power generation just before MER, the LS joint generates substantial power. At BR, the stride leg produces lateral shear peak GRFs, the stride ankle produces minimal power likely contributing to the creation of a stable base, and the LS joint generates substantial power to effectively move energy into the throwing arm.

**Figure 14:**
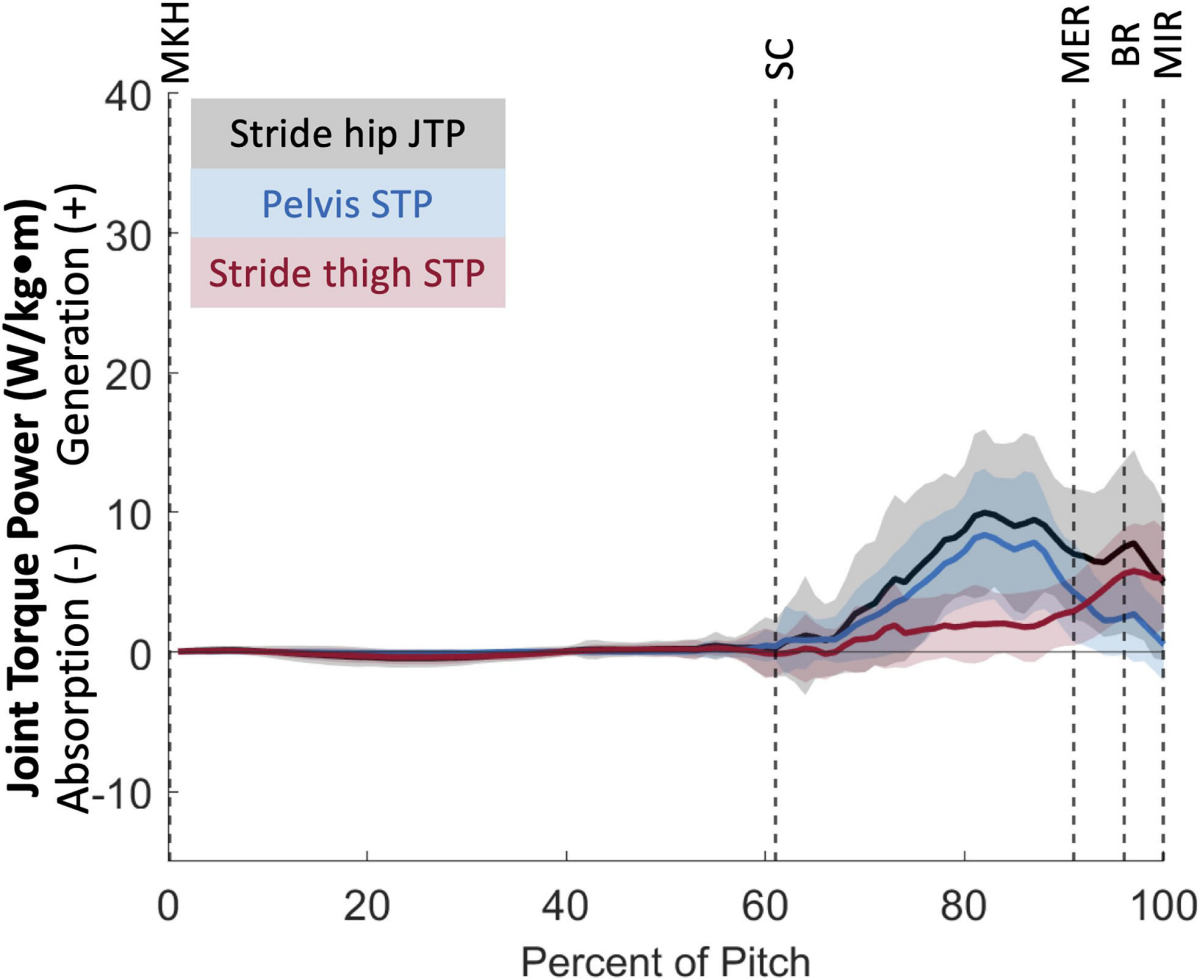
Mean ± SD joint torque power for the stride hip (black) and its components of pelvis segment torque power (blue) and stride thigh segment torque power (red) for the duration of the pitch from maximum knee height (MKH) to maximum internal rotation (MIR).

We can further identify the directionality of energy flow by partitioning joint torque power (JTP) into individual components of proximal segment torque power (STP_p_) and distal segment torque power (STP_d_). As defined in Robertson and Winter [12], the sign and magnitude of STP components indicate the direction of energy flow from generation or absorption by the structures surrounding the joint. In this youth pitcher cohort, two joints appear to have large power generation contributions: the stride hip and the lumbosacral joint. In partitioning JTP at these joints (Figures 10, 14), we see that both the proximal (pelvis) and distal (thigh) STPs are positive, indicating that STP_p_ is generating energy to the proximal segment while STP_d_ is generating energy to the distal segment [12]. At the stride hip, most energy appears to be flowing proximally into the pelvis, moving energy up the kinetic chain, during the arm cocking phase. The structures surrounding the stride hip are also generating energy to the stride thigh likely to create a stable base for the trunk to rotate about. In the arm acceleration and deceleration phases, most energy is flowing distally into the stride thigh, likely to continue stabilization of the center of mass during rapid trunk rotation. At the lumbosacral joint, most energy appears to be flowing proximally into the trunk during the arm cocking phase and at BR. This energy flow likely continues up the kinetic chain to aid in energy generation through the throwing arm. Energy is also flowing into the pelvis at both power generation peaks, which may flow into the stride thigh to aid in creating a stable base.

### Key findings

The main GRF events (peak vertical, propulsive, and braking forces) occur surrounding SC. The main power (energy flow) events follow, with peak stride hip energy generation occurring in the middle of the arm cocking phase and peak lumbosacral energy generation occurring in the arm cocking phase and at BR. This sequence indicates that while the generated propulsive force assists in moving the center of mass toward the home plate, braking force is a larger contributor to energy generation up the kinetic chain. This braking force is likely used by the stride leg to create energy at the stride hip, the most inferior and earliest joint to generate substantial energy. Each subsequent joint up the kinetic chain on both the drive and stride leg generated significantly more JTP than the previous joint, and the lumbosacral joint generated significantly more power than all lower extremity joints. There were no significant differences between timing of peak JTPs with the exception of drive hip JTP occurring significantly later than drive knee JTP. The lack of significant timing differences along with the sign and magnitude of STPs indicate that significant increases in power up the kinetic chain are likely due to the ability of sequentially larger musculature surrounding each joint to generate more power, rather than an additive process of energy transfer between joints. Substantial timing variability in these youth pitchers may indicate a lack of strategy optimization, and these results may differ in more experienced populations who have optimized their strategy for these tightly timed events.

Evidence that hip abductors and adductors become fatigued following a game in collegiate pitchers supports our findings of energy generation at the stride hip, indicating that this joint is important in energy flow up the kinetic chain [32]. Partitioning of energy flow indicates that the stride hip aids in energy flow up the kinetic chain into the lumbosacral joint and in the creation of a stable base upon which the body rotates. Similarly, the lumbosacral joint facilitates energy flow up the kinetic chain using energy generated from the stride thigh along with the trunk musculature generating additional energy.

The lower peak power magnitudes of the drive limb joints along with drive hip extension and external rotation moments during the stride phase support a theory that pitchers do not exclusively use a “drop and drive” or “controlled fall” strategy, but instead use a combination of these two techniques [1]. This combined strategy of the drive leg may be more accurately described as a “controlled drop” with little to no forward drive, as the stride hip reaches peak flexion during the stride phase while extension and external rotation moments work to rotate and translate the center of mass forward in the direction of home plate, but this controlled drop is not coupled with drive limb joint energy generation.

The lumbosacral joint produced the most power during the pitch cycle, supporting the theory that core strength is an important factor in pitch velocity. An analysis of segment powers found that trunk power was a predictor of ball speed [23] in professional and high school pitchers. Our trunk peak power in youth pitchers (36.21 ± 11.32 W/kg) is comparable to trunk peak powers found for professional and high school pitchers in that study (34 ± 14 and 40 ± 11 W/kg, respectively). A high degree of lumbopelvic control has previously been found to be associated with pitch performance, further highlighting the importance of core stability [33]. A study of collegiate-level pitchers agrees with our findings of lumbosacral JTP generation and energy flow proximally into the trunk; however, this study found that pelvis STP was negative in the arm cocking phase [34]. Following the convention of Robertson and Winter [12], these collegiate-level pitchers appear to be transferring energy from the pelvis into the trunk rather than generating energy distally into the pelvis at the lumbosacral joint like our youth pitchers. This is an interesting developmental finding and could indicate that pitchers improve energy flow up the kinetic chain with experience.

### Limitations

There is a large amount of between-subjects variability in this dataset. This is likely due to these young athletes having a lack of experience, leading to an absence of movement strategy self-optimization. While the athlete age range of 9–13 years is an appropriate developmental age bracket, it is also possible that movement strategies differ between the younger and older athletes in the cohort due to years of experience. Additionally, workload data were not collected and may vary considerably between pitchers of this age bracket. Data were normalized to bodyweight (GRFs) or mass^*^height (JTPs) during analysis; however, the more mature athletes in this cohort likely have more muscle mass allowing them to produce more joint power than the younger athletes, which likely influences timing and muscle activation properties of the pitch.

The pitching mound was MLB regulation size, which could be a limitation in this cohort since youth pitchers use a lower mound height. This visual difference may have caused a change in the typical pitch trajectory. However, the mound was adjustable to each pitcher's stride length, aiming to encourage pitchers to use a comfortable and typical technique. Grip tape present on the stride leg force plate is an additional limitation that potentially changes ground reaction forces and mechanics as this does not perfectly replicate competition surfaces.

### Future directions

The results of this paper describe an energy generation strategy used by developmental-aged pitchers. Pitching strategy likely becomes optimized with additional experience, and this analysis could be repeated in high school, college, and professional cohorts to determine if strategy is age dependent, particularly as technology develops and size and strength change dramatically during puberty. This work quantifies the role that the lower extremities and trunk play in proper pitching mechanics. To further apply this work to injury risk, a prospective comparison of the influence of trunk strength and stabilization would help determine specific core strength and neuromuscular control components that are necessary to keep pitchers healthy.

## Data availability statement

The raw data supporting the conclusions of this article will be made available by the authors, upon reasonable request.

## Ethics statement

The studies involving human participants were reviewed and approved by Marquette University Institutional Review Board. Written informed consent to participate in this study was provided by the participants' legal guardian/next of kin.

## Author contributions

MP and MS contributed to the conception and design of the study. MS contributed to data collection. MP performed data processing, analysis, and wrote the first draft of the manuscript. Both authors contributed to manuscript revision, read, and approved the submitted version.

## Conflict of interest

The authors declare that the research was conducted in the absence of any commercial or financial relationships that could be construed as a potential conflict of interest.

## Publisher's note

All claims expressed in this article are solely those of the authors and do not necessarily represent those of their affiliated organizations, or those of the publisher, the editors and the reviewers. Any product that may be evaluated in this article, or claim that may be made by its manufacturer, is not guaranteed or endorsed by the publisher.
